# Liquid Petrolatum and Its Trademark Names

**Published:** 1916-07

**Authors:** 


### LIQUID PETROLATUM AND ITS TRADE-MARK NAMES.

Recently[1] we called attention to the handicap imposed on medical progress by the multiplicity of names for the same therapeutic substance, as well as by unnecessary non-descriptive names. An illustration of the absurd extent to which the multiplication of names can go is furnished in the list of trade appellations given to liquid petrolatum since Sir W. Arbuthnot Lane advocated this product as a remedy for intestinal stasis. About a year ago we published a partial list of names, new and old, for liquid petrolatum.[2] Since then many more new ones have come to notice, among them Bakurol, Interol, Med-O-Lin, Muthol, Semprolin, Whiteruss, Nujol and Stanolax. Nujol is put up by the Standard Oil Company of New Jersey and Stanolax by the Standard Oil Company of Indiana. Probably before long each of the other Standard Oil companies will have its own name for

---

[1] The Nomenclature of Drugs, editorial, *The Journal A. M. A.*, May 29, 1915, p. 1853.

[2] Liquid Petrolatum or "Russion Mineral Oil," Report Council Pharm. and Chem., *The Journal A. M. A.*, May 30, 1914, p. 1740. Adepsine Oil, Amalie, Atoleine, Atolin, Blandine, Crysmalin, Deeline, Glyco, Glycolin, Glymol, Heavy petroleum oil, Liquid Albolene, Liquid Petrolatum, Liquid Saxoline, Mineral Glycerin, Mineral Oil, Neutral-ol, Olo, Paraffin Oil, Paroline, Petralol, Petro, Petrolax, Petrolia, Petronol, Petrosio, Rock Oil, Russian Liquid Petrolatum, Russian Mineral Oil, Russian paraffin oil, Russol, Saxol, Terraline, Terralbolia, Usoline, Water-white mineral oil, White liquid vaseline, White paraffin oil.

liquid petrolatum—that is, if physicians will tolerate it. Proprietary names are coined to sustain the impression, created by advertising, that, in each case, the particular product is superior to all others; exaggerated claims would not be made for the same product under an official title. The clinical investigation conducted under the auspices of the Council on Pharmacy and Chemistry and reported on by Dr. Bastedo[3] showed that the therapeutic results from light and heavy, and from Russian and American liquid petrolatum, are indistinguishable; that the selection of a light or a heavy variety is a matter of individual preference, and that the prime consideration should be to secure a practically odorless and tasteless product. There is no excuse whatever for special brands so far as the medical profession and the public are concerned, but it is otherwise with those who supply the products: More can be charged for a product sold under a trademarked name, for thereby it is possible to make the buyer think that he is getting something better than the official liquid petrolatum. Not a few of those who put out such products under proprietary titles are simply placing their names on liquid petrolatum bought from one of the Standard Oil companies or from some other oil-producing or oil-refining concern. If one prefers the product sold by a particular firm—as Hyson, Westcott & Co., E. R. Squibb & Sons, or any other—let the prescription read, accordingly, Liquid Petrolatum, H., W. & Co., Liquid Petrolatum, Squibb, etc. Liquid Petrolatum U. S. P. should be, and probably is, as pure a product as any on the market. If a given pharmacist does not keep it or if the product he keeps is not practically odorless and tasteless, then physicians should insist that he get a production that conforms to these requirements—or patronize a pharmacist who will do this.—*Journal A. M. A.*

---

[3] Bastedo, W. A.: Clinical Experience with Liquid Paraffin (Liquid Petrolatum), *The Journal A. M. A.*, March 6, 1915, p. 808.

